# Migration Behavior of Technetium-99 in Granite, Clay Rock, and Shale: Insights into Anionic Exclusion Effects

**DOI:** 10.3390/toxics13090760

**Published:** 2025-09-07

**Authors:** Yunfeng Shi, Song Yang, Wenjie Chen, Aiming Zhang, Zhou Li, Longjiang Wang, Bing Lian

**Affiliations:** Department of Nuclear Environmental Science, China Institute for Radiation Protection (CIRP), Taiyuan 030006, China; shiyunfeng@cirp.org.cn (Y.S.);

**Keywords:** granite, clay rock, mudstone shale, anion exclusion, ^99^Tc

## Abstract

One of the key tasks in the geological disposal of radioactive waste is to investigate the blocking ability of different host rocks on nuclide migration in the disposal site. This study conducted experimental and numerical methods to the adsorption, diffusion, and advection–dispersion behavior of ^99^Tc in three types of rocks: granite, clay rock, and mudstone shale, with a focus on the influence of anion exclusion during migration. The research results found that the three types of rocks have no significant adsorption effect on ^99^Tc, and the anion exclusion during diffusion and advection–dispersion processes can block small “channels”, causing some nuclide migration to lag, and accelerate the nuclide migration rate in larger “channels”. In addition, parameters characterizing the size of anion exclusion in different migration behaviors, such as effective diffusion coefficient (*De*) and immobile liquid region porosity ($\theta_{im}$), were fitted and obtained.

## 1. Introduction

Radioactive waste is a unique byproduct generated during the use of atomic energy, and its safe disposal has always been a focal point of public concern. According to the IAEA safety standards, radioactive wastes are categorized into six groups based on their radioactivity levels, ranging from highest to lowest: high-level waste (HLW), intermediate-level waste (ILW), low-level waste (LLW), very low-level waste (VLLW), very short-lived waste (VSLW), and exempt waste (EW) [1]. With the exception of short-lived waste and EW, all other types of waste must undergo various disposal methods, such as near-surface disposal, intermediate-depth disposal, and deep geological repository, for long-term management. The migration of radioactive nuclides from the waste to the surrounding environment must be effectively impeded and isolated through a “multi-barrier system” [2]. Therefore, evaluating the barrier capabilities of different rock types against nuclides is a crucial task in the selection and construction of radioactive waste disposal facilities [3].

Currently, numerous studies have conducted experiments and numerical simulations on the migration of radioactive nuclides through adsorption, diffusion, and convection–dispersion in various types of rocks, yielding a substantial amount of data [4,5,6,7]. In recent years, the research focus has transitioned from determining key parameters of nuclide migration behavior to comprehending the primary factors and mechanisms involved in these processes. For instance, Lee investigated the diffusion behavior of Se (IV) in Tamusu clay rocks, emphasizing the impact of compaction density on diffusion behavior [8]. Sun examined the adsorption and diffusion behavior of Se (IV) and Se (VI) in clay rocks, exploring the influence of different valence states of the same nuclide on their migration behavior [9]. Qi delved into the migration and adsorption behavior of Se (IV) and Sr (II) in granite minerals and fracture fillers, and scrutinized the influence of different chemical forms on the migration behavior of nuclides [10].

In the past, many scholars previously categorized anionic nuclides such as ^99^Tc (TcO_4_^−^), ^129^I (IO_3_^−^), ^36^Cl (Cl^−^), and ^3^H (H^+^) as non-adsorbed nuclides. Consequently, in numerous hydrogeological experiments, researchers tend to employ anionic elements as tracers to derive geological parameters [11,12], overlooking the “accelerated” migration of nuclides due to anionic exclusion. While some studies have addressed the impact of anionic exclusion on the diffusion and convection–dispersion migration behavior of elements like Cl^−^ and IO_3_^−^ [13,14,15], there has been no comprehensive investigation into the effects of anionic exclusion on the adsorption, diffusion, and convection–dispersion of a specific anion nuclide. In 2022, Shi examined the convection–dispersion migration behavior of various iodine ion forms in granite, emphasizing the influence of different chemical forms (I^−^/I_3_^−^) on anionic exclusion [16]. This study offers insights into understanding the “accelerated migration” behavior of anionic nuclides.

^99^Tc is a significant nuclide found in the waste produced by nuclear power plants, boasting a high fission yield (6.13%) in reactors. Compounds of Tc can display oxidation states ranging from -I to VII, typically existing as TcO_4_^−^ in aqueous solutions [17]. Research on the migration behavior of ^99^Tc has primarily centered on the impact of changes in nuclide valence states on solubility [18,19] and anionic exclusion [20,21], driven by its unique characteristics of “multiple chemical valence states” and “non-adsorption.” The former elucidates how redox conditions in the environment influence the migration of ^99^Tc in complex settings, aiding in the separation and elimination of ^99^Tc from multi-nuclide mixed solutions. The latter explores the effects of anionic exclusion on ^99^Tc migration by comparing its behavior with that of other nuclides in various solid-phase media [22].

Diffusion and advection–dispersion are the primary mechanisms through which radioactive nuclides migrate in the rock medium of disposal sites, influenced by physical and chemical factors. Physical factors pertain to the characteristics of nuclide movement channels within the solid phase medium, defined by parameters like porosity, effective diffusion coefficient, and dispersibility [23]. Chemical factors involve the interaction between nuclides and the surrounding media during migration, particularly in terms of adsorption, with characteristic parameters including distribution coefficient and adsorption rate [24]. The migration of nuclides is concurrently affected by physical and chemical factors. To elucidate the specific role of an influencing factor in nuclide migration, it is typically necessary to initially conduct diffusion and advection–dispersion tests using non-adsorbed nuclides (e.g., HTO) and subsequently introduce designated nuclides to investigate the influencing factors of nuclide migration behavior [25,26].

This study utilized batch experiments [27], diffusion experiments [28], and column experiments [29] to replicate the adsorption, diffusion, and advection–dispersion characteristics of ^99^Tc in granite, clay rock, and shale. The essential parameters governing the diffusion and advection–dispersion behavior of ^99^Tc were determined through numerical model fitting [30]. A comparison with the diffusion and advection–dispersion parameters of HTO in the same rock medium facilitated a discussion on the impact of anion repulsion on the migration behavior of ^99^Tc.

## 2. Theory of Adsorption, Diffusion, and Advection–Dispersion

### 2.1. Distribution Coefficient Model

The distribution coefficient is a parameter that characterizes the distribution of nuclides in the solid–liquid phase. According to the IAEA definition, the distribution coefficient is determined by the ratio of nuclide activity concentration in the solid phase to that in the liquid phase [30]. The expression formula is as follows:

(1) $$K_{d}=\frac{(c_{0}-c_{e})\times V}{c_{e}\times m}$$

where $c_{0}$ is the initial activity concentration of nuclides in the liquid phase (Bq/L), $c_{e}$ is the activity concentration of nuclides in the liquid phase at adsorption equilibrium (Bq/L), *V* is the total volume of the solution (mL), and *M* is the mass of the solid phase medium (g).

### 2.2. Diffusion Model

According to the theory of porous media, rock samples are generalized as isotropic homogeneous porous media, and the diffusion behavior of nuclides in the sample is equivalent to one-dimensional. The diffusion equation is established based on the principle of conservation of mass and the concept of typical unit cells, and the obtained diffusion curve is fitted to the solution of the equation to obtain the diffusion coefficient [31].

(2) $$\frac{\partial C}{\partial t}=\frac{D_{a}}{R}\frac{\partial^{2}C}{\partial x^{2}}$$

(3) $$D_{a}=\frac{D_{e}}{\alpha }$$

(4) $$\alpha =\theta +\rho_{b}\times k_{d}$$

(5) $$\theta =1-\frac{\rho_{b}}{\rho_{s}}$$

The initial conditions and boundary conditions are as follows:

Boundary conditions: *C*(*x*,0) *=* 0, *x >* 0

Initial conditions: *C*(0,*t*) *= C*_0_, *t >* 0

(6) $$C\left(\infty ,t\right)=0,\;t\geq 0$$

where *C*(*x*,*t*) is the concentration distribution of tracer in the vertical direction of the thin slice (Bq/mL), $D_{e}$ is the effective diffusion coefficient of the tracer in the vertical direction of the thin film (m^2^/s), $D_{a}$ is the apparent diffusion coefficient of the tracer in the vertical direction of the thin film (m^2^/s), *α* is the capacity factor, *x* is the one-dimensional diffusion distance (cm), *t* is the diffusion time (d), *C_0_* is the initial concentration value of each tracer in the source solution tank (Bq/mL), *K_d_* is the adsorption distribution coefficient of the nuclide (mL/g), θ  is the total porosity of the porous medium, which is the ratio of the pore volume to the total volume of the medium, $\rho_{b}$ is the dry density of the solid phase medium (kg/m^3^), and $\rho_{s}$ is the particle density (kg/m^3^*)*.

### 2.3. Advection–Dispersion Model

#### 2.3.1. Equilibrium Transport (E-T)

The one-dimensional equation is as follows [32]:

(7) $$\frac{\partial }{\partial t}\left(\theta c_{r}+\rho_{b}s\right)=\frac{\partial }{\partial x}\left(\theta D\frac{\partial c_{r}}{\partial x}-J_{w}c\right)-\theta \mu_{l}c_{r}-\rho_{b}\mu_{s}s+\theta r_{l}\left(x\right)+\rho_{b}r_{s}\left(x\right)$$

where $c_{r}$ is the volume-averaged or resident concentration of the liquid phase (*ML^−3^*); *s* is the concentration of the adsorbed phase (*MM^−1^*); *D* is the dispersion coefficient (*L^2^T^−1^*); θ is the volumetric water content (*L^3^L^−3^*); $J_{w}$ is the volumetric water flux density (*LT^−1^*); $\rho_{b}$ is the soil bulk density (*ML^−3^*); $\mu_{l}\;$and $\mu_{s}$ are the first-order decay coefficients for the degradation of the solute in the liquid and adsorbed phases, respectively (*T^−1^*); $r_{l}\;$(*ML^−3^ T^−1^*) and$\;r_{s}$ (*MM^−1^ T^−1^*) are the zero-order production terms for the liquid and adsorbed phases, respectively; *x* is the distance (*L*); and *t* is the time (*T*).

The solute adsorption by the solid phase is expressed using a linear isotherm as follows:

(8) $$s=K_{d}c_{r}$$

where $K_{d}\;$ is an empirical distribution constant (*M^−1^L^3^*). Using (8) and assuming a steady-state flow in a homogeneous soil, (7) may be rewritten as follows:

(9) $$R\frac{\partial c_{r}}{\partial t}=D\frac{\partial^{2}c_{r}}{\partial x^{2}}-v\frac{\partial c_{r}}{\partial x}-\mu c_{r}+r\left(x\right)$$

where $v(=\frac{J_{w}}{\theta }$) is the average pore-water velocity; *R* is the retardation factor given by $R=1+\frac{\rho_{b}K_{d}}{\theta }$; and μ and r are combined first- and zero-order rate coefficients: $\mu =\mu_{l}+\frac{\rho_{b}K_{d}\mu_{s}}{\theta }$, $r(x)=r_{l}(x)+\frac{\rho_{br_{s}(x)}}{\theta },respectively.$

#### 2.3.2. Two-Region Nonequilibrium Transport (T-N)

A two-region transport model was proposed, and it was assumed that the liquid phase can be partitioned into mobile (flowing) and immobile (stagnant) regions (which are produced by anion exclusion). The solute exchange between the two liquid regions is simulated as a first-order kinetic process. The two-region solute transport model is given by [33].

(10) $${(\theta }_{m}+f\rho_{b}K_{d})\frac{\partial c_{m}}{\partial t}{=\theta }_{m}D_{m}\frac{\partial^{2}c_{m}}{\partial x^{2}}-J_{w}\frac{\partial c_{m}}{\partial x}-a\left(c_{m}-c_{im}\right)-\left(\theta_{m}\mu_{l,m}+f\rho_{b}K_{d}\mu_{s,m}\right)c_{m}+\theta_{m}r_{l,m}\left(x\right)+f\rho_{b}r_{s,m}(x)$$

(11) $${(\theta }_{im}+\left(1-f\right)\rho_{b}K_{d})\frac{\partial c_{im}}{\partial t}=a\left(c_{m}-c_{im}\right)-\left(\theta_{im}\mu_{l,im}+\left(1-f\right)\rho_{b}K_{d}\mu_{s,im}\right)c_{im}+\theta_{im}r_{l,im}\left(x\right)+\left(1-f\right)\rho_{b}r_{s,im}\left(x\right)$$

where the subscripts *m* and *im* refer to the mobile and immobile liquid regions, respectively; $J_{w}=v\times \theta =v_{m}\times \theta_{m}$ is the volumetric water flux density; f represents the fraction of adsorption sites that equilibrates with the mobile liquid phase; and a is the first-order mass transfer coefficient governing the rate of solute exchange between the mobile and immobile liquid regions. θ is equal to $\theta_{m}+\theta_{im}$; $\mu_{l,m}$ and $\mu_{l,im}$ are first-order decay coefficients for the mobile and immobile liquid phases, respectively; $\mu_{s,m}$ and $\mu_{s,im}$ are first-order decay coefficients for the mobile and immobile adsorbed phases, respectively; $r_{l,m}$ and $r_{l,im}$ are zero-order production for the mobile and immobile liquid phases, respectively; and ${\;r}_{s,m}$.and $r_{s,im}$ are zero-order production terms for the mobile and immobile adsorbed phases, respectively.

## 3. Experiments

### 3.1. Rock

The granite, clay rock, and mudstone utilized in this study were sourced from radioactive waste disposal sites under construction, planned construction, and existing sites in northwest, northern, and southwestern China, respectively. Following retrieval, all the rock samples were crushed, washed thrice with deionized water, and dried at 100 ± 10 °C for 24 h before being stored. Both batch and column experiments were conducted. Batch experiments and diffusion experiments were conducted using rock samples with a particle size of 0.075 mm or less. In the column experiment, the particle size of the granite was less than 0.075 mm, while the particle size of the clay rock and mudstone shale ranged from 0.425 mm to 0.25 mm.

### 3.2. Rock Sample Analysis

X-ray Powder diffractometer (XRD,D-Power, Anhui Guoke Instrument Technology Co., Ltd., Hefei, China), X-ray Fluorescence Spectrometer (XRF, Zetium, Malvern Panalytical, Malvern, UK), and Scanning Electron Microscopy–Energy-Dispersive Spectrometer (SEM-EDS,TH-F120, CIQTEK Co., Ltd., Hefei, China) were utilized to analyze the mineral and chemical composition of the granite, clay rock, and mudstone shale, respectively.

### 3.3. Batch Experiment

The adsorption behavior was investigated using a batch experiment method. A 1.0 g treated rock sample was weighed and placed in a 15 mL centrifuge tube. Subsequently, a 10 mL nuclide solution (initial activity concentrations of 800 Bq, 1600 Bq, 2400 Bq, 3200 Bq, 4000 Bq) mixed with ultrapure water was added, maintaining a solid–liquid ratio of 1:10 g/mL and a pH value of 7.3–8.0. The mixture was shaken continuously at 25 °C for 48 h to ensure full contact of the sample. Following this, a centrifuge was used to separate the solid and liquid phases. The water sample was then extracted to measure the nuclide’s activity and calculate the activity concentration. The ^99^Tc sample (China Tongfu Co., Ltd., Nantong, China) was analyzed and measured using an ultra-low background liquid scintillation spectrometer (SIM-MAX LSA3000, Shanghai Xinman Sensing Technology Research and Development Co., Ltd., Shanghai, China).

### 3.4. Diffusion Experiment

#### 3.4.1. Diffusion Experimental Device

**Figure 1:**
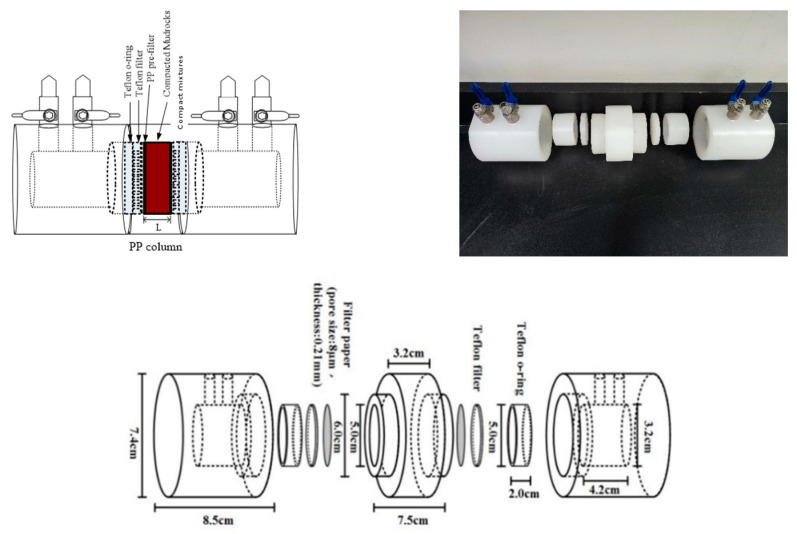
Diffusion experimental device.

Figure 1 illustrates the equipment utilized in the diffusion experiment, comprising three main components: the source liquid tank, the liquid collection tank, and the rock slice. Introduce nuclides into the source liquid tank, fill the liquid tank with ultrapure water devoid of solutes, secure the rock sheet using a porous filter, and position it at the center of the diffusion device using two “O” rings. Additionally, to maintain the long-term integrity of the sample medium and prevent damage, secure the sample with two polyethylene filters on both sides and insert it into the experimental apparatus. The experimental setup parameters are detailed in Table 1.

#### 3.4.2. Diffusion Experimental Procedure

a. Granite, clay rock, and mudstone with a particle size less than 0.075 mm are compacted to obtain experimental samples with a density of 2.0 g/cm^3^, and fixed through filters before being placed in a diffusion tank.

b. Water saturation treatment is performed on rock fragments by filling the source liquid tank with ultrapure water. The water is allowed to permeate into the extraction tank under the influence of a water level difference, saturating the rock fragments with water.

c. After saturating the rock fragments with water, the nuclide HTO (20 Bq/mL) is initially introduced into the source liquid tank. This tank is linked to an external storage tank via a peristaltic pump (30–60 min per cycle, with a water flow rate of 500–1000 mL). The solubility of nuclides in the source liquid tank is maintained at a constant level by continuously monitoring the HTO content in the storage tank. Simultaneously, ultrapure water is added to the extraction tank to ensure a consistent water level on both sides. Periodically, all the solutions in the extraction tank are extracted for HTO activity measurement. The diffusion experiment of HTO is concluded once analysis and calculations confirm the attainment of equilibrium. An ultra-low background liquid scintillation spectrometer (SIM-MAX LSA3000, Shanghai Xinman Sensing Technology Research and Development Co., Ltd.) is employed for the analysis and quantification of HTO (China Tongfu Co., Ltd.).

d. After rinsing the HTO clean, the ^99^Tc (15 Bq/mL) solution is placed into the source tank and connected to an external storage tank using a peristaltic pump (30–60 min per cycle, with a water flow rate of 500–1000 mL). The ^99^Tc content in the storage tank is continuously measured to ensure constant solubility of nuclides in the source tank. All the solutions are regularly taken out from the tank, and the activity of nuclides is measured. After confirming diffusion equilibrium through analysis and calculation, all diffusion tests are stopped.

### 3.5. Column Experiment

#### 3.5.1. Column Experimental Device

**Figure 2:**
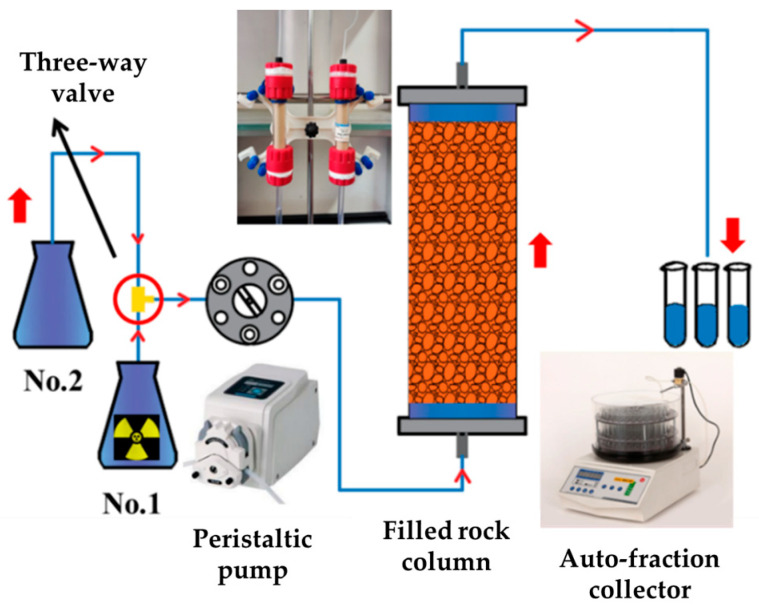
Column experimental device.

The device is shown in Figure 2 and consists of three parts: the injection end, column, and collection end. The injection end includes bottle 1 (for storing nuclide solution) and bottle 2 (for storing ultrapure water). The injection of nuclides is achieved by controlling a three-way valve connected to a peristaltic pump. The column is filled with rock and soil samples, and filters are installed at the upper and lower ends to prevent sample loss. The collection end is used for collecting the effluent from the column, and the automatic collector completes the sample sampling at regular intervals. The device parameters are shown in Table 2.

#### 3.5.2. Column Experimental Procedure

a. Fill the processed granite, clay rock, and mudstone samples into glass columns with a density of 1.25 g/cm^3^. Install filter screens at the upper and lower ends of the column to prevent the loss of soil samples during water flow erosion.

b. To saturate the rock column with water, adjust the three-way valve and open the peristaltic pump to slowly inject ultrapure water from the bottom of the column in bottle 2. Stop when water flows out from the outlet to fully saturate the rock and soil column.

c. Inject HTO solution into bottle 1, adjust the three-way valve, and open the peristaltic pump to inject HTO (5000 Bq) into the soil column instantly. Then, regulate the three-way valve to introduce ultrapure water into the column at a flow rate of 0.25 mL/min while keeping the pH within the range of 7.3–8.0. Periodically collect and analyze samples at the outflow end. Cease the HTO penetration test once the HTO content in the outflow falls below the lower limit of detection.

d. After rinsing the HTO in bottle 1, replace it with ^99^Tc solution and inject ^99^Tc (5000 Bq) into the soil column instantly following the same steps. Collect samples at the outflow end and analyze them periodically. Terminate the test when the nuclide content in the outflow is below the detection lower limit.

### 3.6. Mathematical Model and Parameter Estimations

Use 1stOpt 6.0 (First Optimization) software and STANMOD 2.10 (STudio of ANalytic MODels), respectively, to fit the experimental data obtained from diffusion experiments and convection–dispersion experiments. 1stOpt is a comprehensive mathematical optimization analysis tool software package that has achieved good results in handling nonlinear regression, curve fitting, and other problems through the use of the Universal Global Optimization (UGO) algorithm. STANMOD is a Windows-based computer software package used to evaluate solute transport in porous media using analytical solutions of the advection–dispersion solute transport equation. The software includes modified and updated versions of the CXTFIT code for estimating solute transport parameters using nonlinear least squares parameter optimization methods. Both software use root mean square error (RMSE) to represent the degree of fitting, which is defined as follows:

(12) $$RMSE=\sqrt{\frac{\sum_{i}^{N}{\left(C_{p}-C_{e}\right)}^{2}}{N}}$$

where *C_p_* denotes the results of the numerical fitting and *C_e_* denotes the experimental data.

## 4. Results and Discussion

### 4.1. Mineral Composition and Chemical Composition

**Figure 3:**
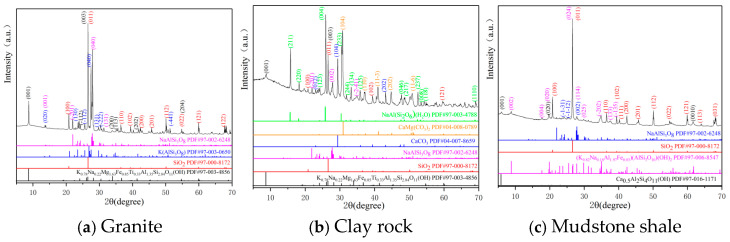
XRD analysis results of rock samples.

**Figure 4:**
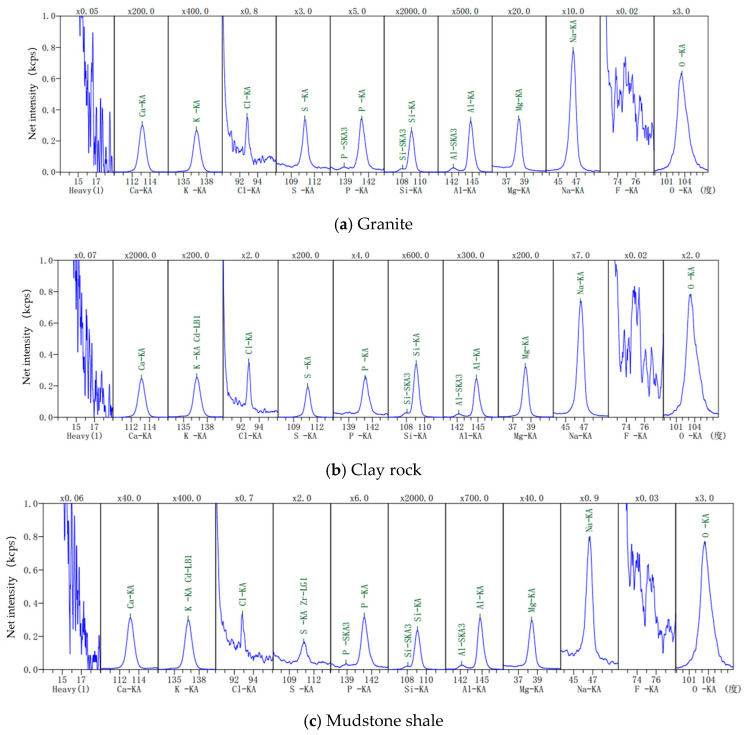
XRF analysis results of rock samples.

The mineral and chemical composition analysis of the granite, clay rock, and mudstone shale is depicted in Figure 3 and Figure 4, and Table 3.

### 4.2. Batch Experiment Results

**Figure 5:**
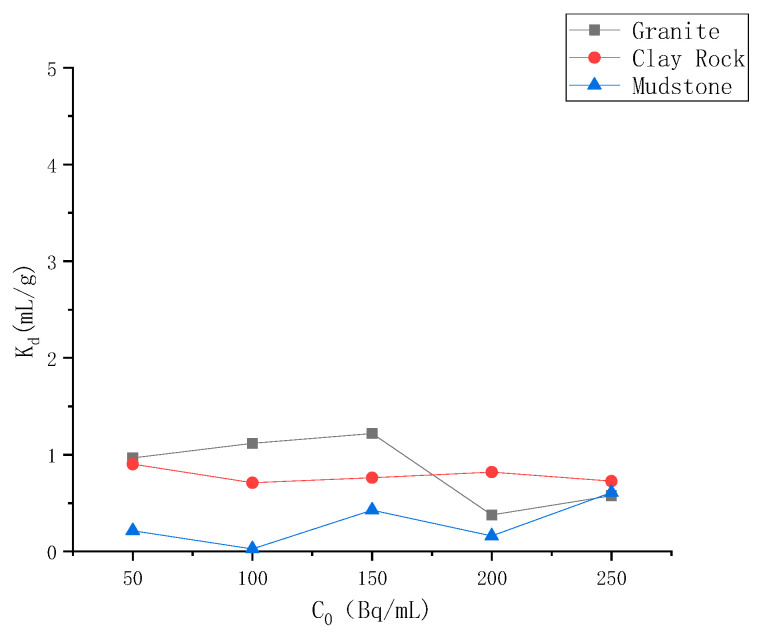
Adsorption capacity of different rocks for ^99^Tc.

Batch experiments were carried out to investigate the adsorption behavior of ^99^Tc in the granite, clay rock, and mudstone shale. The results are illustrated in Figure 5. The distribution coefficients of ^99^Tc for the granite, clay rock, and mudstone shale are 0.85 mL/g, 0.78 mL/g, and 0.30 mL/g, respectively. The solid-phase adsorption capacity in this study was determined by subtracting the liquid phase content post-adsorption from the total input amount, considering measurement errors in the liquid phase sample and solute losses during operations. Subsequent diffusion and convection–dispersion experiments indicated that the granite, clay rock, and mudstone shale exhibited negligible adsorption capacity for ^99^Tc in this investigation.

### 4.3. Diffusion Experiment Results

#### 4.3.1. Diffusion Experiment Results of HTO

**Figure 6:**
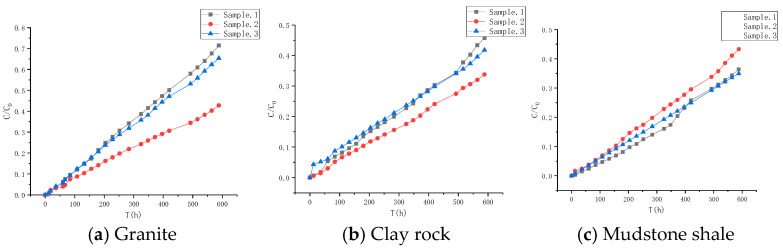
Diffusion curves of HTO in different rocks.

**Figure 7:**
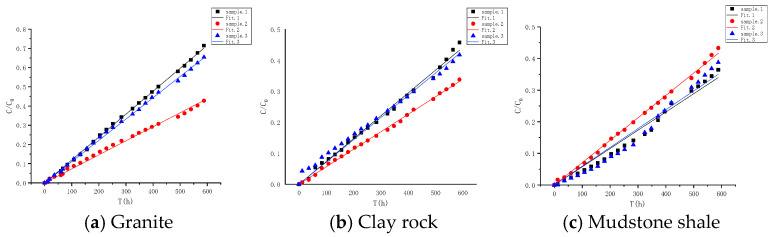
Fitting of diffusion curves of HTO in different rock media.

To examine the diffusion pathways within the granite, clay rock, and mudstone shale, experiments were conducted to analyze the diffusion characteristics of tritiated water (HTO) within them. The experimental findings are shown in Figure 6. Each material was tested in triplicate, with each test lasting 588 h. Following the initiation of the experiments, the diffusion profiles of HTO in all the substrates exhibited a swift escalation. The diffusion capacity of HTO in the three rock types follows the order granite > clay rock > mudstone shale. The diffusion parameters were determined through numerical simulation methods, and the fitting effect, along with related parameters, is presented in Figure 7 and Table 4. The experimental results demonstrate variations in porosity among different rock types: granite (*Ø* = 0.26) > mudstone shale (*Ø* = 0.20) > claystone (*Ø* = 0.18). Regarding the effective diffusion coefficient, a notable discrepancy exists within the granite samples, with values ranging from 2.48 × 10^−12^ to 4.05 × 10^−11^ m^2^/s. In contrast, the disparity between clay rock and mudstone shale is insignificant, with diffusion coefficients ranging from 2.60 × 10^−11^ to 1.99 × 10^−11^ m^2^/s and 2.03 × 10^−11^ to 2.50 × 10^−11^ m^2^/s, respectively.

#### 4.3.2. Diffusion Experiment of ^99^Tc

**Figure 8:**
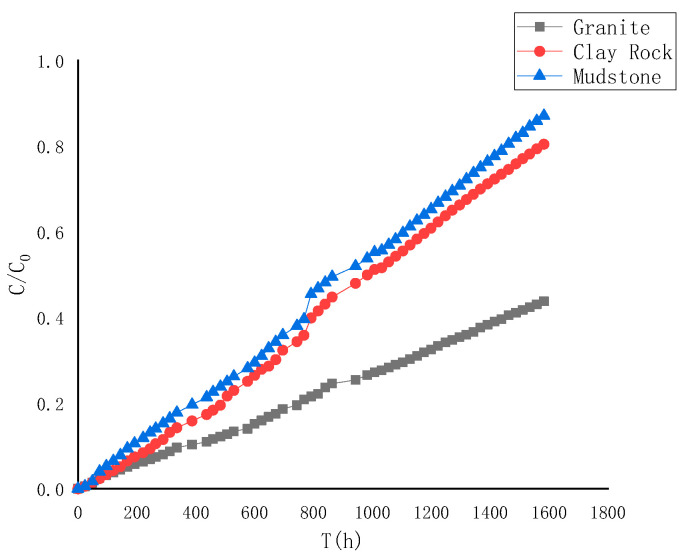
Diffusion curves of ^99^Tc in different rocks.

The ^99^Tc diffusion curve is depicted in Figure 8. Generally, the diffusion pattern of ^99^Tc resembles that of HTO, suggesting negligible adsorption impact of the medium on ^99^Tc.

The diffusion experiment lasted for 1584 h, and the cumulative concentration ratio of ^99^Tc in different rock media was granite < clay rock < mudstone shale, which contrasts significantly with the diffusion behavior of HTO. This difference is attributed to ^99^Tc existing in an anionic state (TcO_4_^−^) in aqueous solution, leading to repulsion by anions in the rock pores. This repulsion results in the blockage of small pores while accelerating the migration of nuclides in larger pores. Moreover, a higher clay mineral content intensifies the repulsive effect, indicating that ^99^Tc diffuses most rapidly in mudstone shale and slowest in granite.

For the fitting of the diffusion curve of ^99^Tc, the “anion repulsion” effect, not explicitly defined in current diffusion models, influences both the distribution coefficient (represented by negative values) and the effective diffusion coefficient (leading to a significant decrease). These parameters directly impact the capacity factor *α* and porosity *Ø* in the fitting process. To enhance the precision of the fitting parameters, the primary approach involves initially determining the apparent diffusion coefficient *Da* and capacity factor *α* through curve fitting. Subsequently, the effective diffusion coefficient *De* is calculated, with the fixed porosity *Ø* remaining constant at its original value and the distribution coefficient set at 0. This method consolidates all the anion repulsion effects within the effective diffusion coefficient.

**Figure 9:**
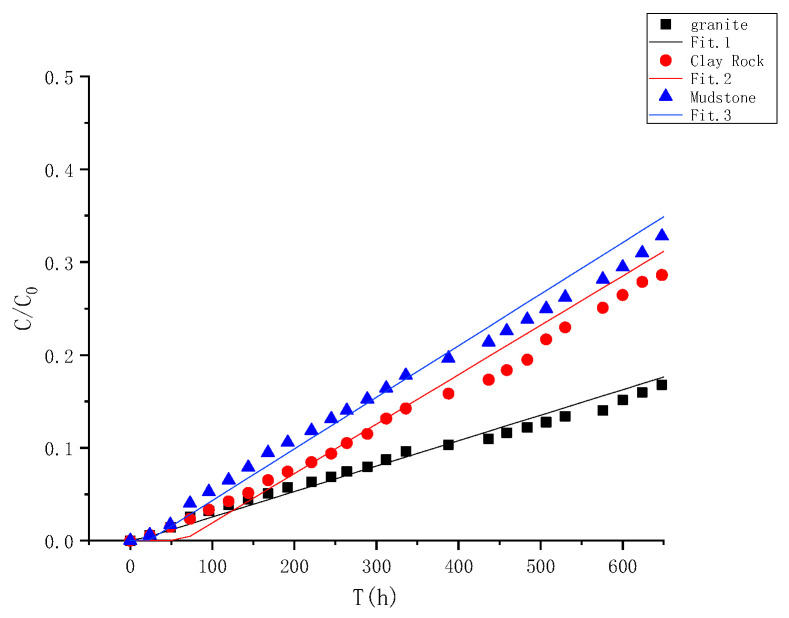
Fitting of diffusion curves of ^99^Tc in different rock media.

The results of the fitting are displayed in Figure 9 and Table 5. Upon comparing the parameters across various rock types, it was observed that the apparent diffusion coefficient follows the order: mudstone > clay rock > granite. Similarly, the order for mud shale > clay rock > granite remains consistent. Regarding the effective diffusion coefficient, the sequence mudstone shale > clay rock > granite was noted, suggesting that the “anion repulsion” of ^99^Tc leads to an overall “accelerated” diffusion of nuclides. Notably, a stronger “anion repulsion” corresponds to a smaller effective diffusion coefficient in these parameters.

### 4.4. Advection–Dispersion Experiment of ^99^Tc

#### 4.4.1. Advection–Dispersion Experiment Results of HTO

**Figure 10:**
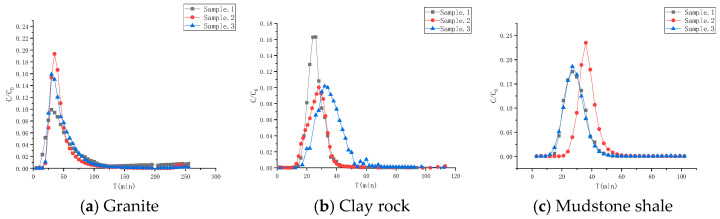
Advection–dispersion curves of HTO in different rocks.

The experimental results are depicted in Figure 10. Overall, the advection–dispersion migration curves of HTO in different rocks exhibit an “S” shape, consistent with the advection–dispersion curve under the instantaneous input mode of non-adsorbed solutes in homogeneous media. To ensure future accuracy in determining relevant physical migration parameters of the medium, three parallel samples are analyzed for each case. The HTO experiment in granite columns lasted 255 min, with peak occurrences at 30, 35, and 30 min, and corresponding peak values of 0.094, 0.193, and 0.159, respectively. For clay rock columns, the experimental durations were 96 min, 103 min, and 97 min, with peak values observed at 24, 28, and 32 min, with corresponding peak values of 0.163, 0.100, and 0.100, respectively. In shale columns, the experimental duration of HTO was 102 min, with peak values recorded at 27, 36, and 27 min, with corresponding peak values of 0.175, 0.235, and 0.190, respectively.

**Figure 11:**
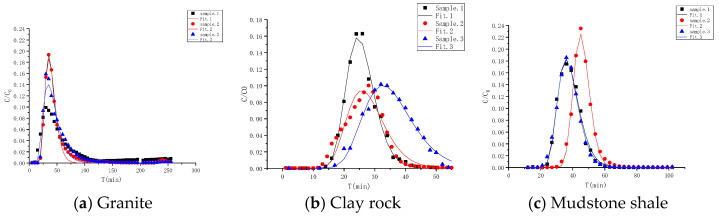
Fitting of advection–dispersion curves of HTO in different rock media.

The advection–dispersion parameters were determined through a balanced model fitting. The fitting effect and associated parameters are presented in Figure 11 and Table 6, respectively. The dispersion values for granite range from 0.631 to 0.640 cm, for clay rock from 0.248 to 0.285 cm, and for mudstone from 0.263 to 0.323 cm.

#### 4.4.2. Advection–Dispersion Experiment Results of ^99^Tc

**Figure 12:**
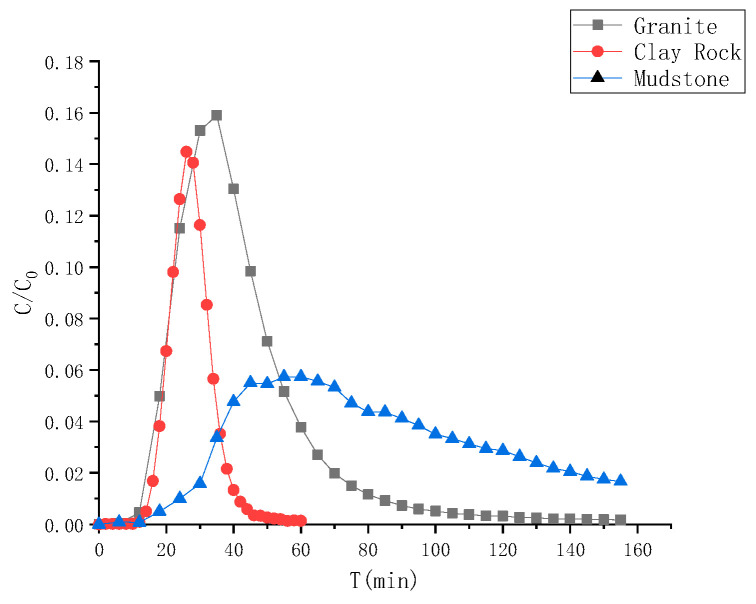
Advection–dispersion curves of ^99^Tc in different rocks.

The advection–dispersion behavior of ^99^Tc in the granite, clay rock, and mudstone shale is illustrated in Figure 12. The migration curve of ^99^Tc in granite and clay rock exhibits an “S” shape, while the migration curve of mudstone shows a distinct “tailing” phenomenon. This observation is attributed to the “anion repulsion” of ^99^Tc during the convection–dispersion process in mudstone. This phenomenon causes a more pronounced “blockage” of small pores in mudstone compared to granite and clay rock, leading to delayed outflow of certain nuclides and the emergence of a “tailing” effect.

**Figure 13:**
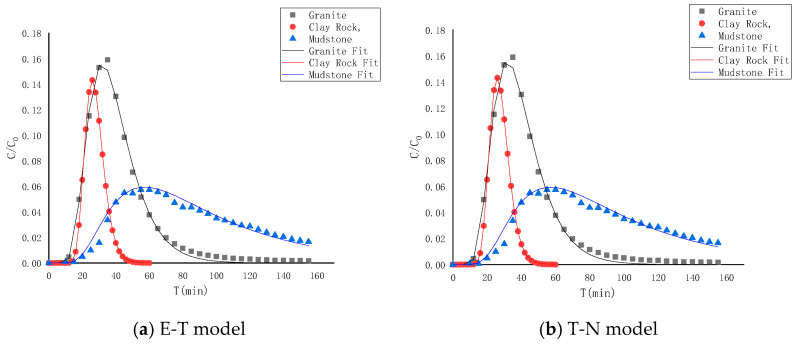
Fitting of advection–dispersion curves of ^99^Tc in different rock media.

Different numerical models were utilized to fit the ^99^Tc advection–dispersion migration curves in granite, clay rock, and mudstone shale. The fitting outcomes and associated parameters are presented in Figure 13 and Table 7. Initially, the equilibrium transport (E-T) model was applied to fit the advection–dispersion migration curves of ^99^Tc in various rocks, demonstrating a satisfactory fitting effect. However, the calculated distribution coefficients were consistently negative, which contradicted the actual conditions. This discrepancy suggests that ^99^Tc experiences “anion repulsion” during migration, leading to some channels being “blocked”, resulting in negative distribution coefficients derived from the equilibrium transport model. Subsequently, the two-region nonequilibrium transport (T-N) model was employed to fit the curves and derive the relevant transfer parameters. The dispersion, movable zone porosity, and immovable zone porosity of ^99^Tc in granite migration are 0.630 cm, 0.26 cm, and 0.11 cm, respectively; in clay rock, they are 0.215 cm, 0.16 cm, and 0.15 cm, respectively; and in mudstone, they are 0.320 cm, 0.17 cm, and 0.18 cm, respectively. Comparing the immovable zone porosity proportions, it was observed that the “anion repulsion” effect influences the ^99^Tc migration behavior in mudstone in the following order: (51%) > clay rock (48%) > granite (30%).

## 5. Conclusions

A systematic study was conducted on the adsorption, diffusion, and advection–dispersion behavior of ^99^Tc in granite, clay rock, and mudstone shale, with a focus on the influence of “anion repulsion” on the migration process of ^99^Tc. The key parameters were obtained through numerical fitting to quantify their degree of influence. The research conclusions are as follows:

1. Batch experiments were conducted, revealing that granite, clay rock, and mudstone shale exhibited negligible adsorption of ^99^Tc.

2. The anion exclusion observed in the diffusion of ^99^Tc can impede certain narrow channels, demonstrating that a higher anion exclusion leads to a reduced effective diffusion coefficient. Conversely, the anion exclusion can enhance the diffusion rate of ^99^Tc in wider water channels. Moreover, the anion exclusion encountered during the diffusion of ^99^Tc is more pronounced in shale than in clay or granite.

3. ^99^Tc is influenced by anion exclusion in the advection–dispersion process, making it challenging to achieve satisfactory fitting results using the equilibrium transport model. Therefore, employing a two-region nonequilibrium transport model becomes essential to accurately determine parameters such as porosity in the immobile zone due to anion exclusion across various media.

## Figures and Tables

**Table 1:** Diffusion experimental device parameters.

Device	Length (cm)	Diameter (cm)
Diffusion device	20.2 cm	7.4 cm
Source liquid tank (liquid collection tank)	4.2 cm	3.2 cm
rock slice	0.3 cm	5 cm

**Table 2:** Column experimental device parameters.

Device	Length	Diameter
Granite column (Particle size < 0.075 mm)	10 cm	1.6 cm
Clay rock column (0.425–0.25 mm)	10 cm	1.6 cm
Mudstone shale column (0.425–0.25 mm)	10 cm	1.6 cm

**Table 3:** Mineral composition and chemical analysis results of rock samples (%).

Rock	Mica	Quartz	Potassium Feldspar	Plagioclase	Illite	Montmorillonite	Zeolite
Granite	10.8	20.5	30.2	38.5	/	/	/
Clay rock	11.1	4.0	/	16.9	/	/	23.6
Mudstone shale	/	46.7	/	9.7	24.6	19.0	/
Rock	Calcite	Dolomite	SiO_2_	Al_2_O_3_	Na_2_O	K_2_O	CaO
Granite	/	/	66.81	15.88	3.83	4.82	3.43
Clay rock	9.6	34.8	30.61	9.72	3.08	2.14	27.95
Mudstone shale	/	/	61.15	20.81	0.33	5.15	0.67
Rock	Fe_2_O_3_	MgO	TiO_2_	P_2_O_5_	SO_3_	MnO	SrO
Granite	3.04	1.24	0.469	0.146	0.10	0.05	0.05
Clay rock	6.74	14.72	0.621	0.080	3.74	0.20	0.27
Mudstone shale	8.47	2.14	0.88	0.16	0.03	0.08	0.01
Rock	BaO	Rb_2_O	ZrO_2_	ZnO	La_2_O_3_	V_2_O_5_	Cr_2_O_3_
Granite	0.05	0.02	0.01	0.01	0.01	0.01	0.004
Clay rock	/	0.02	0.02	0.01	/	0.02	0.021
Mudstone shale	0.02	0.03	0.03	/	0.01	0.02	0.020
Rock	Ga_2_O_3_	PbO	Co_3_O_4_	Cl	CeO_2_	CuO	Er_2_O_3_
Granite	0.003	0.003	0.002	0.032	/	/	/
Clay rock	0.002	/	0.003	0.083	0.006	0.005	0.004
Mudstone shale	0.003	/	0.003	0.023	/	/	/

**Table 4:** Parameters for fitting diffusion curves of HTO in different rock media.

Rock	*Ø*	*α*	*D_a_*	*D_e_*	*R^2^*	*RMSE*
Granite-1	0.26	0.26	1.56 × 10^−10^ m^2^/s	4.05 × 10^−11^ m^2^/s	0.999	0.060
Granite-2	0.26	0.26	9.54 × 10^−11^ m^2^/s	2.48 × 10^−12^ m^2^/s	0.998	0.094
Granite-3	0.26	0.26	1.45 × 10^−10^ m^2^/s	3.76 × 10^−11^ m^2^/s	0.999	0.067
Clay rock-1	0.18	0.18	1.42 × 10^−10^ m^2^/s	2.60 × 10^−11^ m^2^/s	0.998	0.096
Clay rock-2	0.18	0.18	1.09 × 10^−10^ m^2^/s	1.99 × 10^−11^ m^2^/s	0.999	0.042
Clay rock-3	0.18	0.18	1.38 × 10^−10^ m^2^/s	2.53 × 10^−11^ m^2^/s	0.998	0.161
Mudstone shale-1	0.20	0.20	1.02 × 10^−10^ m^2^/s	2.03 × 10^−11^ m^2^/s	0.994	0.172
Mudstone shale-2	0.20	0.20	1.25 × 10^−10^ m^2^/s	2.50 × 10^−11^ m^2^/s	0.999	0.063
Mudstone shale-3	0.20	0.20	1.05 × 10^−10^ m^2^/s	2.10 × 10^−11^ m^2^/s	0.989	0.260

**Table 5:** Parameters for fitting diffusion curves of ^99^Tc in different rock media.

Rock	*Ø*	*α*	*D_a_* (m^2^/s)	*D_e_* (m^2^/s)	*K_d_* (mL/g)	*R^2^*	*RMSE*
Granite	0.26	0.26	3.59 × 10^−11^	9.34 × 10^−12^	0	0.999	0.006
Clay rock	0.18	0.18	8.97 × 10^−11^	1.71 × 10^−11^	0	0.998	0.014
Mudstone shale	0.20	0.20	9.28 × 10^−11^	1.86 × 10^−11^	0	0.997	0.014

**Table 6:** Parameters for fitting advection–dispersion curves of HTO in different rock media.

Rock	*V*	*D*	*D_L_*	*R*	*RMSE*
Granite-1	0.241 cm/min	0.152 cm^2^/min	0.631 cm	1.00	5.25 × 10^−6^
Granite-2	0.242 cm/min	0.155 cm^2^/min	0.640 cm	1.00	3.89 × 10^−6^
Granite-3	0.242 cm/min	0.154 cm^2^/min	0.636 cm	1.00	9.45 × 10^−5^
Clay rock-1	0.383 cm/min	0.095 cm^2^/min	0.248 cm	1.00	1.57 × 10^−5^
Clay rock-2	0.358 cm/min	0.095 cm^2^/min	0.269 cm	1.00	3.46 × 10^−10^
Clay rock-3	0.322 cm/min	0.092 cm^2^/min	0.285 cm	1.00	9.26 × 10^−6^
Mudstone shale-1	0.342 cm/min	0.098 cm^2^/min	0.287 cm	1.00	1.04 × 10^−5^
Mudstone shale-2	0.312 cm/min	0.101 cm^2^/min	0.323 cm	1.00	1.24 × 10^−5^
Mudstone shale-3	0.346 cm/min	0.091 cm^2^/min	0.263 cm	1.00	9.14 × 10^−6^

**Table 7:** Parameters for fitting advection–dispersion curves of ^99^Tc in different rock media.

Model	Granite Column					
E-T	*V*	*D*	*D_L_*	*R*	*K_d_*	RMSE
	0.254 cm/min	0.16 cm^2^/min	0.630 cm	0.981	<0	1.97 × 10^−5^
T-N	*V*	*D*	*D_L_*	$\theta_{m}$	$\theta_{im}$	RMSE
	0.254 cm/min	0.16 cm^2^/min	0.630 cm	0.26	0.11	1.10 × 10^−5^
Model	Clay rock column					
E-T	*V*	*D*	*D_L_*	*R*	*K_d_*	RMSE
	0.256 cm/min	0.055 cm^2^/min	0.215 cm	0.707	<0	1.60 × 10^−5^
T-N	*V*	*D*	*D_L_*	$\theta_{m}$	$\theta_{im}$	RMSE
	0.256 cm/min	0.055 cm^2^/min	0.215 cm	0.16	0.15	1.45 × 10^−5^
Model	Mudstone shale column					
E-T	*V*	*D*	*D_L_*	*R*	*K_d_*	RMSE
	0.286 cm/min	0.092 cm^2^/min	0.320 cm	0.954	<0	1.14 × 10^−5^
T-N	*V*	*D*	*D_L_*	$\theta_{m}$	$\theta_{im}$	RMSE
	0.286 cm/min	0.092 cm^2^/min	0.320 cm	0.17	0.18	1.07 × 10^−5^

## Data Availability

The raw data supporting the conclusions of this article wil be made available by the authors on request.
